# Hijacking the Pathway: Perspectives in the Treatment of Mature T-cell Leukemias

**DOI:** 10.1097/HS9.0000000000000573

**Published:** 2021-06-01

**Authors:** Linus Wahnschaffe, Marco Herling

**Affiliations:** 1 Department I of Internal Medicine, Center for Integrated Oncology (CIO), Aachen-Bonn-Cologne-Duesseldorf, University of Cologne (UoC), Germany; 2 Excellence Cluster for Cellular Stress Response and Aging-Associated Diseases (CECAD), University of Cologne, Germany; 3 Center for Molecular Medicine Cologne (CMMC), University of Cologne, Germany; 4 Department of Hematology, Cellular Therapy and Hemostaseology, University of Leipzig, Germany

## Introduction

The most prevalent post-thymic T-cell malignancies of primary leukemic presentation in Western countries are T-cell prolymphocytic leukemia (T-PLL) and T-cell large granular lymphocyte leukemia (T-LGL). Each, T-PLL and T-LGL, are diagnosed at incidences of approximately 1.1 per million per year in individuals of median ages of 65 and 55 years (yet with broad ranges), respectively.^1–3^ Both malignancies represent extremes of the spectrum of T-cell differentiation and growth kinetics, hence have “opposing” natural clinical courses. The typically CD4^+^ memory-type T-PLL cell is functionally inert, but highly proliferative. Exponentially increasing tumor burden involves blood, (failing) bone marrow (BM), and spleen. The median overall survival (OS) is 2–3 years. On the contrary, T-LGL cells are typically aberrant CD8^+^ cytotoxic T-lymphocytes that underly highly symptomatic auto-immune phenomena in an otherwise low proliferative disease (median OS ~10 years).^3^ Related to T-LGL is the even rarer chronic lymphoproliferative disorder of NK-cells, which too, presents with the characteristic cytopenias.

The different biology and clinical presentations of T-PLL and T-LGL call for different treatment strategies with respect to targets and therapeutic intensities. In the following, we will discuss future approaches for both malignancies that are based on our current molecular disease concepts as well as on encouraging preclinical and early clinical data. Not discussed here are the other 2 forms of mature T-cell leukemias, namely the endemic adult T-cell leukemia/lymphoma and the dermotropic Sézary Syndrome.

### Problems with the current therapeutic approaches

Common to T-PLL and T-LGL is our limited armamentarium of disease-specific therapeutics that induce clonal eradication or sustained tumor control. There is no European Medicines Agency (EMA) or U.S. Food and Drug Administration (FDA) approved drug for these indications. In T-PLL, first-line therapy should include the monoclonal CD52-antibody alemtuzumab that is available through a compassionate-use program. The initial overall response rates after alemtuzumab as a single agent or in combination with chemotherapeutics are ~90%, but all patients eventually relapse. Conventional chemotherapeutics, such as purine analogs, are second-line attempts. A consolidating allogeneic stem cell transplantation (alloHSCT) at first best response is the only current option for long-term disease control with a 3-year median OS of transplanted patients between 21% and 40%,^4^ but only about one third of T-PLL patients are eligible to undergo this procedure.

**Figure 1.**
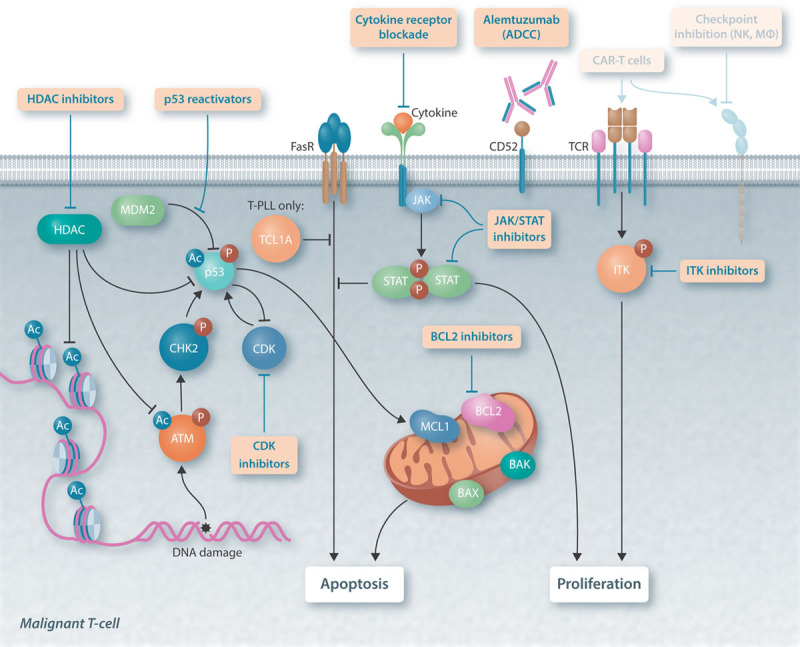
**Currently recognized key pathways in T-PLL and T-LGL that provide potential targets for novel treatment strategies.** Illustrated are receptors and molecular signaling nodes as well as their interactions in alignment with the modes of action of most promising compound classes in the treatment of T-PLL and T-LGL. A defective response to DNA damage caused by dysfunctional ATM is a pathogenetic hallmark of T-PLL. Re-activation of p53 or downstream modification of the BCL2/MCL1 equilibrium provide reasonable approaches to restore the tumor cell’s ability to undergo intrinsic apoptosis. Further strategies aim to inhibit vital growth signals, that is, via inhibition of proliferation and differentiation signals induced by cytokine or TCR signaling. Preliminary efforts (displayed transparent) are undertaken in the field of immunotherapies: CAR-T cells targeting the TCR β-chain are under development. The use of immune checkpoint inhibitors and modulation of the non-T-immune cell synapse (eg, NK-cells, macrophages [MΦ]) is effective in other T-cell malignancies, but has thus far not been evaluated systematically in T-PLL or in T-LGL. Ac = acetylated; ADCC= antibody-dependent cell-mediated cytotoxicity; ATM = ataxia-telangiectasia mutated; BAK = BCL2 antagonist/killer; BAX = BCL2 associated X; BCL2 = B-cell lymphoma 2; CAR-T = chimeric antigen receptor T-cells; CDK = cyclin-dependent kinase; CHK2 = checkpoint kinase 2; FasR = FAS cell surface death receptor; HDAC = histone deacetylase inhibitors; ITK = IL2 inducible T-cell kinase; JAK = janus kinase; MCL1 = myeloid cell leukemia 1; MDM2 = mouse double minute 2; NK = natural killer cells; P = phosphorylated; STAT = signal transducer and activator of transcription; TCL1A = T-cell leukemia/lymphoma 1A; T-LGL = T-cell large granular lymphocyte leukemia; T-PLL = T-cell prolymphocytic leukemia; TCR = T-cell receptor.

**Figure 2.**
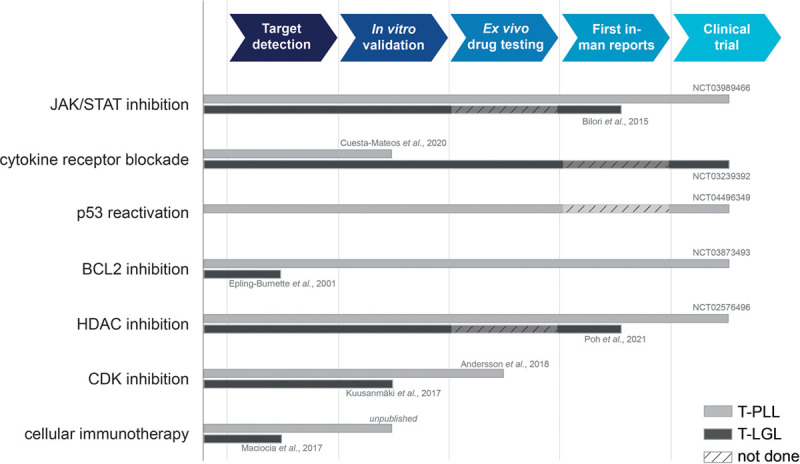
**Current state of development of different targeted therapy approaches in T-PLL and T-LGL.** Most promising substance classes are displayed with their progression in the developmental process, from identification of a potential target, through in-vitro validation of its functional relevance, ex-vivo drug testing, first application in single r/r patients, to conduction of a clinical trial. Grey bars represent the progress of the substance class in T-PLL, black bars indicate its current state in T-LGL. References next to the bars indicate the respective clinical trial registration number or most advanced publication. Hatched areas illustrate steps in the developmental process on which no data were published. BCL2 = B-cell lymphoma 2; CDK = cyclin-dependent kinase; HDAC = histone deacetylase; JAK = janus kinase; r/r = relapsed/refractory; STAT = signal transducer and activator of transcription; T-LGL = T-cell large granular lymphocyte leukemia; T-PLL = T-cell prolymphocytic leukemia.

Although the prognosis is better for T-LGL (10-y OS ~70%),^3^ therapeutic options in this entity have hardly changed over the past 2 decades. Besides supportive measures (eg, hematopoietic growth factors), causal therapies act anti-proliferative and immunosuppressive to alleviate cytopenias and to reduce (eg, arthritic) symptoms. A phase II comparative analysis of the first-line sequences of methotrexate followed by cyclophosphamide and vice versa characterizes our current state of innovation in T-LGL (NCT01976182). Commonly used second-line substances include cyclosporin A, fludarabine, or bendamustine.^3^ Alemtuzumab (NCT00345345) as well as other monoclonal antibodies (eg, siplizumab [NCT00123942, anti-CD2] or Mik-beta-1 [NCT00076180], anti-CD122) were investigated in pilot series without having shown substantial benefits thus far.

Recent genetic profiling studies and their implications on deregulated molecular pathways have advanced our disease concepts of T-PLL and T-LGL. In conjunction with compound sensitivity screens, this improved biological understanding has ushered an era of intensified interrogations of pathway-specific novel substances, which are outlined here (Figures 1 and 2).

### Inhibition of JAK/STAT and cytokine signaling

The janus kinase (JAK)/signal transducer and activator of transcription (STAT) pathway is the key transducer of cytokine and other growth signals in T-lymphocytes, mediating cell proliferation, differentiation, and migration. Activating mutations of *JAK/STAT* family genes are frequent in T-PLL; for example, *JAK3* (36.4%), *STAT5B* (18.8%), and *JAK1* (6.3%). In combination with losses of negative regulators of JAK/STAT signaling, found in 71.4% of cases, a total of about 90% of T-PLL carry a genetic lesion that can be implicated in the ubiquitously enhanced STAT5B phosphorylation in T-PLL cells.^5^ In T-LGL, constitutive activation of STAT3 is often accompanied by *STAT3* gain-of-function mutations (~40% of patients).^6^ Recurrent *STAT5B* mutations are highly prevalent in the rare subset of CD4^+^ T-LGL.^7^

Ruxolitinib, a selective JAK1/2 inhibitor, was among the top-25 active substances in a library screening of more than 300 approved drugs in 39 T-PLL cases.^8^ Ruxolitinib combined with tofacitinib (JAK2/3 inhibitor) indicated modest activity in 2 relapsed/refractory (r/r) T-PLL patients.^9^ Regimens of JAK inhibitors in combination with other substance classes are currently under investigation. Single centers reported encouraging responses of r/r T-PLL patients to the combination of B-cell lymphoma 2 (BCL2) antagonists and JAK inhibitors.^10^ The combination of itacitinib (JAK1 inhibitor) and alemtuzumab is currently evaluated in a phase I trial (NCT03989466).

Specific next-generation STAT3/5 inhibitors provide a promising approach to overcome the limitations of upstream-targeting by JAK antagonists. Inhibitors of STAT5’s SH2-domain or its N-terminus represent such strategies,^11^ which are currently being studied in our laboratory.

The C-C chemokine receptor type 7 (CCR7), which mediates T-cell migration and invasion, is expressed on the majority of T-PLL cells. Anti-T-cell-leukemic activity of a monoclonal anti-CCR7 antibody was observed in preclinical in vitro and in vivo studies.^12^

In T-LGL, ruxolitinib and tofacitinib have been applied to patients with related rheumatoid arthritis. While improvement of the synovitis findings was observed in ~89% of patients, these compounds provided only moderate hematologic improvements.^13^ Promising results were observed for BNZ-1 (Bioniz), a multi-cytokine inhibitor that targets the γ-chain receptor subunits of interleukin (IL)-2, IL-9, and IL-15. In primary T-LGL cells, BNZ-1 (Bioniz) induced decreases of STAT3 phosphorylation upon IL-15 treatment and a marked reduction of cytokine-mediated cell survival.^14^ First clinical data with BNZ-1 in LGL are developed in an ongoing phase I/II trial (NCT03239392).

### P53 reactivation and targeting of the BCL2 family

Protection from programmed cell death is a hallmark of T-PLL and T-LGL. Although both malignancies share the virtual absence of deletions or mutations of the tumor suppressor gene *TP53*, the mechanisms that underlie this cell-death resistance differ. In T-PLL, the tumor cells carry incompetent damage repair mechanisms due to genetically determined hypomorphic ataxia-telangiectasia mutated (ATM).^2^ They are primed for apoptosis due to replicative, oxidative, or other stresses, but cannot execute the ATM-CHK2-P53 axis. As a consequence, the p53 protein remains in a predominantly inactivated state, bound to its inhibitor mouse double minute 2 (MDM2) and with hypo-phosphorylated and deacetylated activation patterns.^2^ We demonstrated that MDM2 antagonists and histone deacetylase inhibitors (HDACi) provide an efficient strategy to derepress p53 and reinstate apoptotic competence.^2^ Several MDM2/4 antagonists (eg, nutlin derivatives) and HDACi clustered among the 20 most efficient substances in a drug screening study, and their combination showed synergistic and specific potency in primary T-PLL cells.^2,8^ An upcoming phase IIa trial will investigate the MDM2 inhibitor APG-115 (Ascentage Pharma Group) as a single agent and in combination with the BCL-2 inhibitor APG-2575 (Ascentage Pharma Group) in r/r T-PLL patients (NCT04496349).

Downstream of p53, members of the BCL2 superfamily (eg, BCL2, BCL-XL, myeloid cell leukemia 1 [MCL1], BCL2 associated X, BCL2 antagonist/killer) that regulate mitochondrial apoptosis, provide additional therapeutic targets. The BCL2-antagonist venetoclax showed high in vitro activity.^8^ However, several small case series indicate that in r/r T-PLL venetoclax as a single agent does not impose good tumor control; for example, median OS of 32.5 days from start of treatment.^15^ Synergistic effects of venetoclax with inhibitors of ITK, JAK/STAT, or HDAC, are reported from preclinical tests.^10,16^ First encouraging clinical data derive from combinations of venetoclax with bendamustine, with pentostatin, with ibrutinib (see also phase II trial NCT03873493), or with MCL1 inhibitors.^15,17–19^

In T-LGL, alterations of the BCL2/MCL1 equilibrium have been reported as well and were linked to overactivated STAT3. To our knowledge, there is no published data on the efficacy of therapeutic BCL2-family modulation in T-LGL. Combined use of JAK/STAT inhibitors and Bcl-2 homology domain 3 (BH3) mimetics might provide a rationale-based strategy.

In addition to dysfunctional intrinsic apoptosis, extrinsic, activation-induced apoptosis pathways are altered in T-PLL and T-LGL as well. Resistance towards Fas cell surface death receptor (FAS)-mediated cell death is well established in T-LGL and was recently also observed in T-PLL cells.^16,20^ In T-PLL, this reduced susceptibility to FAS-induced apoptosis is enhanced by overexpression of the TCL1A oncogene^16^; in T-LGL it is linked to constitutive activation of JAK/STAT and Ras/Raf pro-survival signaling.^21^ Following promising in vitro data,^22^ the Ras inhibitor tipifarnib showed high toxicity and weak responses in T-LGL patients.^23^

### Epigenetic deregulations as specific targets?

In T-PLL, recurrent mutations of genes such as *TET2*, *DNMT3A*, *EZH2*, *BCOR,*^2^ in combination with highly promising sensitivities towards HDAC inhibitors,^2,8,24^ underline the relevance of epigenetic modifications. Studies on global patterns of DNA methylation and histone modulations are underway. Clinical data from 8 r/r T-PLL patients indicate that cladribine (purine analogue with “epigenetic activity”), with or without additional HDAC inhibition, might resensitize towards alemtuzumab.^25^ Tinostamustine (EDO-S101; Mundipharma-EDO GmbH), a covalent fusion of bendamustine and vorinostat (HDACi), demonstrated very promising preclinical activity in vitro and in mice,^26^ and was granted Orphan Drug Designation status by the FDA and EMA. The trial NCT02576496 evaluates its activity in T-PLL.

Epigenetic dysregulations were also identified in T-LGL. Recurrent downregulation of SOCS3, a negative regulator of JAK/STAT signaling, was counteracted with demethylating agents, leading to a decrease of pSTAT3 in primary T-LGL cells.^27^ Treatment of an r/r T-LGL patient with belinostat (HDACi) resulted in a marked BM recovery for more than 15 months.^28^

### A role for targeting cell cycle deregulation?

Pharmacologic inhibition of cell cycle regulators such as cyclin-dependent kinases (CDKs) revealed strong responses in ex vivo screenings of T-PLL cells and in *STAT3*-mutated cell lines.^8,29^ In T-PLL, the high preclinical activity of SNS-032 (Sunesis Pharmaceuticals) (CDK2, CDK7, CDK9 inhibitor) and of LDC526 (Bayer AG) (CDK9 inhibitor) showed associations with myelocytomatosis viral oncogene homolog (MYC) expression levels and inhibited MYC downstream pathways.^8,30^ CDK9 inhibition led to downregulation of the anti-apoptotic protein MCL1, implicating a combined use of CDK inhibitors with BH3 mimetics. To our knowledge, there is no published data on clinical activity of CDK inhibitors in T-PLL or T-LGL.

### Is it time for a cell-based anti-T-cell attack?

It is well known that T-cell malignancies are amenable to the graft-versus-lymphoma effect of an alloHSCT. However, the feasibility of specific immune-cell based therapies, for example, T-cell checkpoint blockade, bispecific antibodies, or chimeric antigen receptor T-cells (CAR-T-cells) in a T-cell tumor is challenged by risks of hyper-progression or fratricide. Therefore, it appears attractive to also modulate non-T immune cell synapses in T-PLL and T-LGL to revert the tolerance of effectors such as macrophages or NK-cells, for example, by inhibition of “do-not-attack-me” signals via blockade of CD47 or KIR3DL2 receptors, respectively.^31,32^

Moreover, the development of CAR-T-cells that target the constant region of the TCR β-chain (TRBC1/2) provides an elegant solution to specifically kill clonal T-PLL or T-LGL cells (individually only expressing either TRBC1 or TRBC2) while preserving a sufficient number of healthy T-cells that express the TRBC that is not present at the malignant T-cells.^33^ First in vitro data indicate a high specificity of anti-TRBC CAR-T cells towards primary T-PLL cells. Overall, we will likely witness preclinical and first clinical data from such strategies of either checkpoint modulation or genetically engineered effector cells in T-cell leukemias in the near future.

## Conclusions

With the aid of high-throughput genomic analyses and other advanced technologies, we have identified a wide range of altered pathways involved in the leukemogenesis of T-PLL and T-LGL, including those that are pivotal to clonal sustenance, hence, representing actionable vulnerabilities. JAK/STAT blockers, BCL2 antagonists, and HDAC inhibitors are currently the most advanced substance classes with promising preclinical and clinical data, particularly in T-PLL. They will likely be further implemented in the treatment of r/r patients within the next years. There also is a wide spectrum of other new substances that show great promise, including immune-modulatory or direct cell-based strategies. It is difficult to anticipate which of these strategies will emerge as a next clinical standard in T-PLL or T-LGL.

It has become obvious that responses to these novel inhibitors are assigned to subgroups of patients rather than being effective across all cases. It has also become evident that strategies to combine agents that target multiple pathways are capable to provide over-additive (synergistic) efficacy while reducing toxicity. For this reason, and to avoid futile testing of single agents in multi-refractory patients, refined computational prediction tools fed by individual data from ex vivo drug assays and molecular profiling are needed.^34^ From this we anticipate that future therapies of T-PLL and T-LGL will likely be heterogeneous, but more personalized. Given the rarity of mature T-cell leukemias, it is now more essential than ever to orchestrate scientific efforts in international collaborations, multidisciplinary consortia, and multi-center clinical trials. Under these conditions and in view of the array of available new therapeutic strategies, we are optimistic that there will be reasonably fast clinical progress in the field of the historically “underrecognized” mature T-cell leukemias.

## Disclosures

The authors have no conflicts of interest to disclose.

## Sources of funding

The European Union supports MH as part of the Transcan-II initiative (ERANETPLL) and as part of EraPerMed JAKSTATTARGET.
